# Bis(4-meth­oxy­benzoato)-κ^2^ *O*,*O*′;κ*O*-bis­(nicotinamide-κ*N* ^1^)zinc(II)

**DOI:** 10.1107/S1600536810032885

**Published:** 2010-08-21

**Authors:** Tuncer Hökelek, Güner Saka, Barış Tercan, Erdinç Tenlik, Hacali Necefoğlu

**Affiliations:** a Department of Physics, Hacettepe University, 06800 Beytepe, Ankara, Turkey; b Department of Chemistry, Hitit University, 19030 Ulukavak, Çorum, Turkey; c Department of Physics, Karabük University, 78050, Karabük, Turkey; d Department of Chemistry, Kafkas University, 63100 Kars, Turkey

## Abstract

The asymmetric unit of the title complex, [Zn(C_8_H_7_O_3_)_2_(C_6_H_6_N_2_O)_2_], contains three crystallographically independent mol­ecules with similar configurations. The Zn^II^ cation is coordinated by two N atoms of two nicotinamide ligands and three O atoms from two 4-meth­oxy­benzoate anions in a distorted trigonal-bipyramidal geometry. In each independent mol­ecule, one Zn—O bond distance [2.5181 (12), 2.5931 (12) and 2.4085 (12) Å for the three mol­ecules] is significantly longer than the other two. In the crystal structure, extensive N—H⋯O and C—H⋯O hydrogen bonding links the mol­ecules into a three-dimensional network. π–π contacts between the pyridine rings and between the pyridine and benzene rings [centroid–centroid distances = 3.7655 (9) and 3.8453 (10) Å, respectively] further stabilize the crystal structure.

## Related literature

Nicotinamide is a form of niacin; for background to niacin, see: Krishnamachari (1974 ▶). For *N*,*N*-diethyl­nicotinamide, see: Bigoli *et al.* (1972 ▶). For related structures, see: Greenaway *et al.* (1984 ▶); Hökelek *et al.* (2009*a* ▶,*b* ▶, 2010*a* ▶,*b* ▶,*c* ▶,*d* ▶).
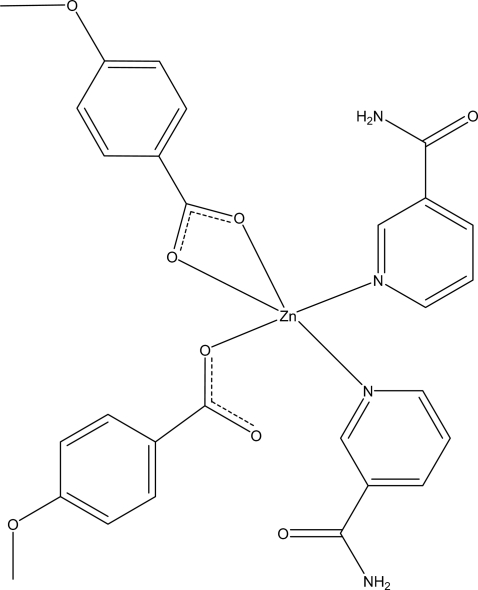


## Experimental

### 

#### Crystal data

[Zn(C_8_H_7_O_3_)_2_(C_6_H_6_N_2_O)_2_]
*M* *_r_* = 611.92
Triclinic, 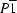

*a* = 10.6828 (2) Å
*b* = 16.6230 (3) Å
*c* = 23.8011 (4) Å
α = 77.050 (2)°
β = 85.654 (3)°
γ = 78.526 (2)°
*V* = 4034.63 (13) Å^3^
*Z* = 6
Mo *K*α radiation
μ = 0.97 mm^−1^
*T* = 100 K
0.26 × 0.24 × 0.20 mm

#### Data collection

Bruker Kappa APEXII CCD area-detector diffractometer
Absorption correction: multi-scan (*SADABS*; Bruker, 2005 ▶) *T*_min_ = 0.776, *T*_max_ = 0.823
72134 measured reflections
20155 independent reflections
14979 reflections with *I* > 2σ(*I*)
*R*_int_ = 0.040

#### Refinement

*R*[*F* ^2^ > 2σ(*F* ^2^)] = 0.037
*wR*(*F* ^2^) = 0.092
*S* = 1.03
20155 reflections
1162 parameters
H atoms treated by a mixture of independent and constrained refinement
Δρ_max_ = 0.42 e Å^−3^
Δρ_min_ = −0.46 e Å^−3^

### 

Data collection: *APEX2* (Bruker, 2007 ▶); cell refinement: *SAINT* (Bruker, 2007 ▶); data reduction: *SAINT*; program(s) used to solve structure: *SHELXS97* (Sheldrick, 2008 ▶); program(s) used to refine structure: *SHELXL97* (Sheldrick, 2008 ▶); molecular graphics: *Mercury* (Macrae *et al.*, 2006 ▶); software used to prepare material for publication: *WinGX* (Farrugia, 1999 ▶) and *PLATON* (Spek, 2009 ▶).

## Supplementary Material

Crystal structure: contains datablocks I, global. DOI: 10.1107/S1600536810032885/xu5015sup1.cif

Structure factors: contains datablocks I. DOI: 10.1107/S1600536810032885/xu5015Isup2.hkl

Additional supplementary materials:  crystallographic information; 3D view; checkCIF report

## Figures and Tables

**Table 1:** Selected bond lengths (Å)

Zn1—O1	2.5181 (12)
Zn1—O2	1.9631 (12)
Zn1—O5	1.9392 (12)
Zn1—N1	2.0793 (15)
Zn1—N3	2.0561 (14)
Zn2—O9	1.9523 (12)
Zn2—O10	2.5931 (12)
Zn2—O13	1.9317 (12)
Zn2—N5	2.0536 (15)
Zn2—N7	2.0669 (14)
Zn3—O17	2.4085 (12)
Zn3—O18	1.9987 (12)
Zn3—O21	1.9436 (12)
Zn3—N9	2.0840 (14)
Zn3—N11	2.0613 (14)

**Table 2:** Hydrogen-bond geometry (Å, °)

*D*—H⋯*A*	*D*—H	H⋯*A*	*D*⋯*A*	*D*—H⋯*A*
N2—H2*A*⋯O24^i^	0.82 (2)	2.16 (2)	2.966 (2)	168.8 (2)
N2—H2*B*⋯O4^ii^	0.84 (2)	2.16 (2)	2.991 (2)	176.6 (2)
N4—H4*A*⋯O17^iii^	0.83 (2)	2.08 (2)	2.903 (2)	169.4 (2)
N4—H4*B*⋯O16^iv^	0.85 (2)	2.59 (2)	3.152 (2)	125.0 (2)
N4—H4*B*⋯O19^v^	0.85 (2)	2.52 (2)	3.225 (2)	141.3 (2)
N6—H6*A*⋯O23^vi^	0.78 (2)	2.40 (2)	3.073 (2)	146 (2)
N6—H6*B*⋯O10^vii^	0.86 (2)	2.11 (2)	2.959 (2)	170.3 (2)
N8—H8*D*⋯O8^iv^	0.79 (2)	2.13 (2)	2.909 (2)	166 (2)
N8—H8*E*⋯O20^viii^	0.87 (2)	2.19 (2)	3.058 (2)	174.8 (2)
N10—H10*A*⋯O12^ix^	0.87 (2)	2.21 (2)	3.077 (2)	172 (2)
N10—H10*B*⋯O15^vi^	0.84 (2)	2.10 (2)	2.917 (2)	162.8 (2)
N12—H12*A*⋯O11^iii^	0.83 (2)	2.55 (2)	3.268 (2)	145 (2)
N12—H12*B*⋯O1^iii^	0.87 (2)	2.07 (2)	2.940 (2)	171.9 (2)

Symmetry codes: (i) 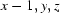; (ii) 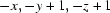; (iii) 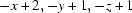; (iv) 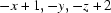; (v) 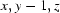; (vi) 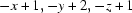; (vii) 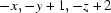; (viii) 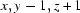; (ix) 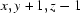.

**Fig. 1.**
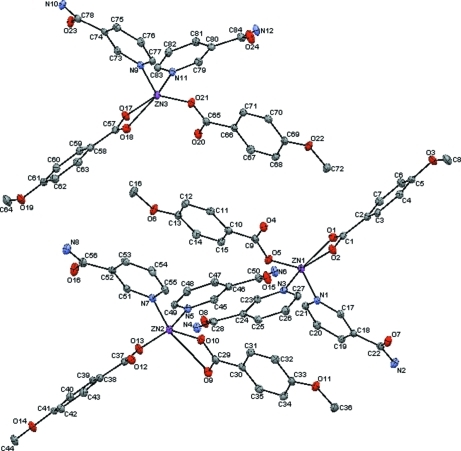
The molecular structure of the title molecule with the atom-numbering scheme. Displacement ellipsoids are drawn at the 40% probability level.

## supplementary crystallographic information

### Comment

As a part of our ongoing investigation on transition metal complexes of nicotinamide (NA), one form of niacin (Krishnamachari, 1974), and/or the nicotinic acid derivative *N*,*N*-diethylnicotinamide (DENA), an important respiratory stimulant (Bigoli *et al.*, 1972), the title compound was synthesized and its crystal structure is reported herein.

The title compound, (I), is a monomeric complex, and its asymmetric unit contains three crystallographically independent molecules. The Zn^II^ centers are coordinated by three O atoms from two 4-methoxybenzoate ligands, which act in different modes-monodentate and bidentate, respectively, and N atoms of the nicotinamide ligands (Fig. 1). So that, all three independent molecules are five-coordinated in distorted trigonal-bipyramidal geometry. The structures of similar complexes of Cu^II^, Co^II^, Ni^II^ and Zn^II^ ions, [Cu(C_8_H_7_O_2_)_2_(C_6_H_6_N_2_O)_2_].2(H_2_O), (II) (Hökelek *et al.*, 2010*a*), [Co(C_8_H_7_O_3_)_2_(C_6_H_6_N_2_O)(H_2_O)_2_], (III) (Hökelek *et al.*, 2010*b*), [Co(C_8_H_7_O_3_)_2_(C_6_H_6_N_2_O)_2_(H_2_O)_2_].2H_2_O, (IV) (Hökelek *et al.*, 2010*c*), [Ni(C_8_H_7_O_3_)_2_(C_6_H_6_N_2_O)_2_(H_2_O)_2_].2H_2_O, (V) (Hökelek *et al.*, 2010*d*), [Zn(C_8_H_8_NO_2_)_2_(C_6_H_6_N_2_O)_2_].H_2_O, (VI) (Hökelek *et al.*, 2009*a*) and [Zn(C_9_H_10_NO_2_)_2_(C_6_H_6_N_2_O)_2_(H_2_O)_2_], (VII) (Hökelek *et al.*, 2009*b*) have also been determined.

In the title compound (Fig. 1), the average Zn—O bond length is 2.1387 (12) Å (Table 1) and the Zn atoms are displaced out of the least-squares planes of the carboxylate groups: Zn1 by -0.0684 (2) Å and -0.1173 (2) Å from (O1/C1/O2) and (O4/C9/O5), Zn2 by -0.0104 (2) Å and 0.1363 (2) Å from (O9/C29/O10) and (O12/C37/O13) and Zn3 by 0.0517 (2) and -0.0728 (2) Å from (O17/C57/O18) and (O20/C65/O21), respectively. The dihedral angles between the planar carboxylate groups and the adjacent benzene rings A (C2—C7), B (C10—C15), E (C30—C35), F (C38—C43), I (C58—C63) and J (C66—C71) are 4.72 (8), 5.90 (15), 1.88 (13), 1.44 (9), 3.66 (9) and 9.14 (12) °, respectively, while those between rings A, B, C (N1/C17—C21), D (N3/C23—C27), E, F, G (N5/C45—C49), H (N7/C51—C55), I, J, K (N9/C73—C77) and L (N11/C79—C83) are A/B = 83.56 (6), A/C = 20.47 (6), A/D = 79.71 (5), B/C = 82.66 (5), B/D = 14.74 (5), C/D = 74.07 (5), E/F = 80.00 (6), E/G = 69.70 (5), E/H = 21.71 (5), F/G = 10.67 (5), F/H = 77.31 (6), G/H = 66.72 (5), I/J = 79.56 (6), I/K = 24.86 (5), I/L = 83.23 (5), J/K = 71.13 (6), J/L = 5.72 (6) and K/L = 72.72 (6) °.

In (I), the O1—Zn1—O2, O9—Zn2—O10 and O17—Zn3—O18 angles are 57.53 (5)°, 56.19 (5) and 59.04 (4)°, respectively. The corresponding O—M—O (where M is a metal) angles are 60.32 (4)° in (III), 59.02 (8)° in (VI), 60.03 (6)° in (VII) and 55.2 (1)° in [Cu(Asp)_2_(py)_2_] (where Asp is acetylsalicylate and py is pyridine) [(VIII); Greenaway *et al.*, 1984].

In the crystal structure, intramolecular C—H···O and intermolecular N—H···O and C—H···O hydrogen bonds (Table 2) link the molecules into a three-dimensional network. The π–π contacts between the pyridine rings and between the pyridine and benzene rings, Cg4—Cg12^i^ and Cg4—Cg2^ii^ [symmetry codes: (i) x - 1, y + 1, z, (ii) 1 - x, 1 - y, 1 - z, where Cg2, Cg4 and Cg12 are the centroids of the rings B (C10—C15), D (N3/C23—C27) and L (N11/C79—C83), respectively] may also stabilize the structure, with centroid-centroid distances of 3.7655 (9) and 3.8453 (10) Å, respectively.

### Experimental

The title compound was prepared by the reaction of ZnSO_4_.H_2_O (1.80 g, 10 mmol) in H_2_O (50 ml) and NA (2.44 g, 20 mmol) in H_2_O (50 ml) with sodium 4-methoxybenzoate (3.48 g, 20 mmol) in H_2_O (100 ml). The mixture was filtered and set aside to crystallize at ambient temperature for one week, giving colorless single crystals.

### Refinement

H atoms of NH_2_ groups were located in difference Fourier maps and refined isotropically. The remaining H atoms were positioned geometrically with C—H = 0.93 and 0.96 Å for aromatic and methyl H atoms, respectively, and constrained to ride on their parent atoms, with *U*_iso_(H) = *xU*_eq_(C), where *x* = 1.5 for methyl H and *x* = 1.2 for aromatic H atoms.

### Crystal data

**Table:**

[Zn(C_8_H_7_O_3_)_2_(C_6_H_6_N_2_O)_2_]	*Z* = 6
*M**_r_* = 611.92	*F*(000) = 1896
Triclinic, *P*1	*D*_x_ = 1.511 Mg m^−^^3^
Hall symbol: -P 1	Mo *K*α radiation, λ = 0.71073 Å
*a* = 10.6828 (2) Å	Cell parameters from 9864 reflections
*b* = 16.6230 (3) Å	θ = 2.5–28.1°
*c* = 23.8011 (4) Å	µ = 0.97 mm^−^^1^
α = 77.050 (2)°	*T* = 100 K
β = 85.654 (3)°	Block, colorless
γ = 78.526 (2)°	0.26 × 0.24 × 0.20 mm
*V* = 4034.63 (13) Å^3^	

### Data collection

**Table:**

Bruker Kappa APEXII CCD area-detector diffractometer	20155 independent reflections
Radiation source: fine-focus sealed tube	14979 reflections with *I* > 2σ(*I*)
graphite	*R*_int_ = 0.040
φ and ω scans	θ_max_ = 28.5°, θ_min_ = 0.9°
Absorption correction: multi-scan (*SADABS*; Bruker, 2005)	*h* = −14→13
*T*_min_ = 0.776, *T*_max_ = 0.823	*k* = −20→22
72134 measured reflections	*l* = −31→29

### Refinement

**Table:**

Refinement on *F*^2^	Primary atom site location: structure-invariant direct methods
Least-squares matrix: full	Secondary atom site location: difference Fourier map
*R*[*F*^2^ > 2σ(*F*^2^)] = 0.037	Hydrogen site location: inferred from neighbouring sites
*wR*(*F*^2^) = 0.092	H atoms treated by a mixture of independent and constrained refinement
*S* = 1.03	*w* = 1/[σ^2^(*F*_o_^2^) + (0.0414*P*)^2^ + 0.1789*P*] where *P* = (*F*_o_^2^ + 2*F*_c_^2^)/3
20155 reflections	(Δ/σ)_max_ = 0.006
1162 parameters	Δρ_max_ = 0.42 e Å^−^^3^
0 restraints	Δρ_min_ = −0.46 e Å^−^^3^

### Special details

**Table:**

Geometry. All esds (except the esd in the dihedral angle between two l.s. planes) are estimated using the full covariance matrix. The cell esds are taken into account individually in the estimation of esds in distances, angles and torsion angles; correlations between esds in cell parameters are only used when they are defined by crystal symmetry. An approximate (isotropic) treatment of cell esds is used for estimating esds involving l.s. planes.
Refinement. Refinement of F^2^ against ALL reflections. The weighted R-factor wR and goodness of fit S are based on F^2^, conventional R-factors R are based on F, with F set to zero for negative F^2^. The threshold expression of F^2^ > 2sigma(F^2^) is used only for calculating R-factors(gt) etc. and is not relevant to the choice of reflections for refinement. R-factors based on F^2^ are statistically about twice as large as those based on F, and R- factors based on ALL data will be even larger.

### Fractional atomic coordinates and isotropic or equivalent isotropic displacement parameters (Å^2^)

**Table:**

	*x*	*y*	*z*	*U*_iso_*/*U*_eq_	
Zn1	0.313492 (18)	0.366125 (12)	0.622308 (9)	0.01588 (6)	
Zn2	0.352863 (19)	0.301859 (12)	1.053489 (9)	0.01722 (6)	
Zn3	0.976026 (18)	1.016176 (12)	0.288848 (9)	0.01588 (6)	
O1	0.39085 (11)	0.33289 (7)	0.72375 (5)	0.0209 (3)	
O2	0.22139 (11)	0.30695 (8)	0.68818 (5)	0.0208 (3)	
O3	0.15524 (12)	0.13733 (8)	0.95436 (5)	0.0250 (3)	
O4	0.17745 (11)	0.52611 (8)	0.62504 (5)	0.0216 (3)	
O5	0.37345 (11)	0.47164 (7)	0.59757 (5)	0.0208 (3)	
O6	0.43183 (11)	0.84998 (7)	0.52950 (5)	0.0204 (3)	
O7	0.25931 (12)	0.53514 (9)	0.40279 (6)	0.0322 (4)	
O8	0.47812 (11)	0.03835 (7)	0.61971 (6)	0.0224 (3)	
O9	0.45314 (11)	0.36051 (8)	0.99141 (5)	0.0223 (3)	
O10	0.27624 (11)	0.35992 (8)	0.94883 (5)	0.0222 (3)	
O11	0.59603 (11)	0.54610 (8)	0.73967 (5)	0.0232 (3)	
O12	0.47687 (12)	0.14356 (8)	1.04057 (5)	0.0236 (3)	
O13	0.28361 (11)	0.19987 (7)	1.07052 (5)	0.0214 (3)	
O14	0.20753 (11)	−0.17617 (7)	1.12763 (6)	0.0227 (3)	
O15	0.20039 (12)	0.63532 (8)	1.02430 (6)	0.0291 (3)	
O16	0.40959 (12)	0.11180 (8)	1.26802 (6)	0.0282 (3)	
O17	1.04363 (11)	0.97596 (8)	0.38711 (5)	0.0210 (3)	
O18	0.87293 (11)	0.95390 (8)	0.35102 (5)	0.0212 (3)	
O19	0.78586 (12)	0.80368 (8)	0.61939 (5)	0.0224 (3)	
O20	0.85139 (11)	1.17047 (7)	0.30187 (5)	0.0217 (3)	
O21	1.04437 (11)	1.11930 (7)	0.26900 (5)	0.0208 (3)	
O22	1.09807 (11)	1.49966 (7)	0.21544 (6)	0.0240 (3)	
O23	0.92177 (12)	1.20096 (8)	0.07362 (6)	0.0262 (3)	
O24	1.12752 (11)	0.68778 (7)	0.28666 (6)	0.0232 (3)	
N1	0.19882 (13)	0.37139 (9)	0.55429 (6)	0.0171 (3)	
N2	0.06975 (16)	0.53483 (11)	0.36749 (7)	0.0219 (4)	
H2A	0.0786 (17)	0.5760 (12)	0.3421 (8)	0.019 (5)*	
H2B	−0.0003 (18)	0.5187 (11)	0.3709 (8)	0.018 (5)*	
N3	0.48010 (13)	0.28705 (9)	0.60648 (6)	0.0158 (3)	
N4	0.69315 (16)	0.00035 (10)	0.61994 (7)	0.0200 (4)	
H4A	0.7647 (18)	0.0139 (11)	0.6166 (8)	0.016 (5)*	
H4B	0.6860 (18)	−0.0506 (13)	0.6320 (8)	0.025 (6)*	
N5	0.19293 (13)	0.38451 (9)	1.07177 (6)	0.0170 (3)	
N6	−0.01415 (17)	0.67577 (11)	1.03019 (8)	0.0244 (4)	
H6A	−0.0070 (19)	0.7202 (13)	1.0129 (9)	0.030 (6)*	
H6B	−0.086 (2)	0.6600 (12)	1.0391 (9)	0.030 (6)*	
N7	0.47047 (13)	0.28584 (9)	1.12176 (6)	0.0176 (3)	
N8	0.60015 (16)	0.10780 (11)	1.30460 (7)	0.0208 (4)	
H8D	0.5895 (18)	0.0675 (12)	1.3282 (9)	0.026 (6)*	
H8E	0.6731 (19)	0.1242 (12)	1.3019 (8)	0.026 (6)*	
N9	0.85885 (13)	1.02845 (9)	0.22012 (6)	0.0168 (3)	
N10	0.72992 (16)	1.20652 (11)	0.03746 (7)	0.0223 (4)	
H10A	0.6560 (19)	1.1910 (12)	0.0412 (8)	0.027 (6)*	
H10B	0.7391 (19)	1.2516 (13)	0.0144 (9)	0.029 (6)*	
N11	1.13843 (13)	0.93611 (9)	0.26882 (6)	0.0153 (3)	
N12	1.34124 (16)	0.64813 (10)	0.27654 (7)	0.0224 (4)	
H12A	1.333 (2)	0.5981 (14)	0.2854 (9)	0.044 (7)*	
H12B	1.418 (2)	0.6591 (12)	0.2761 (9)	0.029 (6)*	
C1	0.29193 (16)	0.30146 (10)	0.73072 (8)	0.0170 (4)	
C2	0.25311 (16)	0.25679 (10)	0.78890 (8)	0.0171 (4)	
C3	0.13826 (16)	0.22777 (10)	0.79820 (8)	0.0175 (4)	
H3	0.0854	0.2353	0.7674	0.021*	
C4	0.10110 (17)	0.18752 (11)	0.85301 (8)	0.0192 (4)	
H4	0.0238	0.1687	0.8588	0.023*	
C5	0.18076 (17)	0.17579 (11)	0.89893 (8)	0.0192 (4)	
C6	0.29574 (17)	0.20519 (11)	0.89017 (8)	0.0217 (4)	
H6	0.3485	0.1978	0.9210	0.026*	
C7	0.33092 (17)	0.24513 (11)	0.83587 (8)	0.0199 (4)	
H7	0.4076	0.2647	0.8303	0.024*	
C8	0.04683 (19)	0.09769 (13)	0.96477 (8)	0.0313 (5)	
H8A	0.0421	0.0703	1.0047	0.047*	
H8B	0.0545	0.0568	0.9413	0.047*	
H8C	−0.0293	0.1392	0.9553	0.047*	
C9	0.28465 (16)	0.53433 (11)	0.60331 (7)	0.0166 (4)	
C10	0.31871 (16)	0.61930 (10)	0.58207 (7)	0.0153 (4)	
C11	0.43776 (16)	0.62713 (11)	0.55511 (7)	0.0169 (4)	
H11	0.4943	0.5795	0.5488	0.020*	
C12	0.47221 (16)	0.70479 (11)	0.53776 (7)	0.0168 (4)	
H12	0.5514	0.7094	0.5195	0.020*	
C13	0.38835 (16)	0.77638 (11)	0.54759 (7)	0.0164 (4)	
C14	0.26949 (16)	0.76976 (11)	0.57452 (8)	0.0177 (4)	
H14	0.2134	0.8174	0.5813	0.021*	
C15	0.23561 (16)	0.69100 (11)	0.59123 (8)	0.0184 (4)	
H15	0.1559	0.6864	0.6088	0.022*	
C16	0.34915 (17)	0.92627 (11)	0.53688 (8)	0.0229 (4)	
H16A	0.3944	0.9721	0.5256	0.034*	
H16B	0.3214	0.9211	0.5766	0.034*	
H16C	0.2762	0.9367	0.5133	0.034*	
C17	0.21054 (16)	0.42806 (11)	0.50529 (8)	0.0178 (4)	
H17	0.2712	0.4618	0.5031	0.021*	
C18	0.13687 (16)	0.43897 (11)	0.45768 (8)	0.0174 (4)	
C19	0.04810 (17)	0.38722 (11)	0.46058 (8)	0.0191 (4)	
H19	−0.0024	0.3923	0.4294	0.023*	
C20	0.03620 (17)	0.32754 (11)	0.51122 (8)	0.0202 (4)	
H20	−0.0216	0.2916	0.5141	0.024*	
C21	0.11107 (16)	0.32237 (11)	0.55697 (8)	0.0189 (4)	
H21	0.1007	0.2836	0.5910	0.023*	
C22	0.16008 (17)	0.50751 (11)	0.40618 (8)	0.0206 (4)	
C23	0.48424 (16)	0.20469 (10)	0.61163 (7)	0.0159 (4)	
H23	0.4084	0.1843	0.6200	0.019*	
C24	0.59610 (16)	0.14823 (11)	0.60509 (7)	0.0155 (4)	
C25	0.70925 (16)	0.17920 (11)	0.59214 (7)	0.0169 (4)	
H25	0.7861	0.1433	0.5873	0.020*	
C26	0.70518 (16)	0.26460 (11)	0.58660 (7)	0.0184 (4)	
H26	0.7795	0.2866	0.5778	0.022*	
C27	0.59038 (16)	0.31657 (11)	0.59423 (7)	0.0169 (4)	
H27	0.5887	0.3736	0.5909	0.020*	
C28	0.58516 (17)	0.05697 (11)	0.61515 (7)	0.0169 (4)	
C29	0.38364 (17)	0.37940 (10)	0.94675 (8)	0.0177 (4)	
C30	0.43568 (16)	0.42607 (10)	0.89192 (7)	0.0160 (4)	
C31	0.36663 (17)	0.44726 (11)	0.84179 (8)	0.0188 (4)	
H31	0.2860	0.4337	0.8430	0.023*	
C32	0.41565 (16)	0.48819 (11)	0.79010 (8)	0.0188 (4)	
H32	0.3682	0.5024	0.7570	0.023*	
C33	0.53688 (17)	0.50788 (10)	0.78834 (7)	0.0163 (4)	
C34	0.60823 (16)	0.48612 (10)	0.83812 (7)	0.0175 (4)	
H34	0.6895	0.4988	0.8368	0.021*	
C35	0.55725 (16)	0.44565 (10)	0.88937 (7)	0.0165 (4)	
H35	0.6047	0.4313	0.9225	0.020*	
C36	0.52520 (18)	0.57312 (12)	0.68812 (8)	0.0254 (4)	
H36A	0.5777	0.5979	0.6571	0.038*	
H36B	0.4508	0.6139	0.6939	0.038*	
H36C	0.4995	0.5257	0.6786	0.038*	
C37	0.36803 (17)	0.13662 (11)	1.06136 (8)	0.0189 (4)	
C38	0.32856 (16)	0.05274 (11)	1.07873 (7)	0.0167 (4)	
C39	0.41094 (17)	−0.01964 (11)	1.07027 (8)	0.0203 (4)	
H39	0.4922	−0.0159	1.0541	0.024*	
C40	0.37452 (17)	−0.09777 (11)	1.08548 (8)	0.0210 (4)	
H40	0.4304	−0.1457	1.0792	0.025*	
C41	0.25362 (17)	−0.10318 (11)	1.11019 (7)	0.0181 (4)	
C42	0.16967 (17)	−0.03088 (11)	1.11905 (8)	0.0194 (4)	
H42	0.0888	−0.0347	1.1357	0.023*	
C43	0.20675 (16)	0.04611 (11)	1.10317 (7)	0.0188 (4)	
H43	0.1502	0.0942	1.1088	0.023*	
C44	0.29057 (18)	−0.25313 (11)	1.12221 (8)	0.0251 (4)	
H44A	0.2447	−0.2987	1.1335	0.038*	
H44B	0.3620	−0.2630	1.1466	0.038*	
H44C	0.3207	−0.2494	1.0828	0.038*	
C45	0.19034 (17)	0.46750 (11)	1.05576 (7)	0.0171 (4)	
H45	0.2657	0.4857	1.0415	0.021*	
C46	0.08131 (17)	0.52752 (11)	1.05929 (7)	0.0178 (4)	
C47	−0.03127 (17)	0.49980 (11)	1.08085 (7)	0.0204 (4)	
H47	−0.1066	0.5380	1.0838	0.025*	
C48	−0.02866 (18)	0.41400 (12)	1.09786 (8)	0.0234 (4)	
H48	−0.1024	0.3941	1.1128	0.028*	
C49	0.08380 (17)	0.35845 (11)	1.09249 (7)	0.0202 (4)	
H49	0.0842	0.3011	1.1036	0.024*	
C50	0.09297 (17)	0.61805 (11)	1.03675 (8)	0.0198 (4)	
C51	0.45678 (16)	0.22669 (11)	1.16912 (8)	0.0177 (4)	
H51	0.3920	0.1962	1.1705	0.021*	
C52	0.53452 (16)	0.20865 (10)	1.21624 (7)	0.0167 (4)	
C53	0.62936 (16)	0.25576 (11)	1.21433 (8)	0.0188 (4)	
H53	0.6828	0.2458	1.2452	0.023*	
C54	0.64308 (17)	0.31808 (11)	1.16553 (8)	0.0213 (4)	
H54	0.7051	0.3508	1.1636	0.026*	
C55	0.56339 (17)	0.33059 (11)	1.12013 (8)	0.0191 (4)	
H55	0.5742	0.3714	1.0872	0.023*	
C56	0.50987 (17)	0.13816 (11)	1.26563 (8)	0.0179 (4)	
C57	0.94215 (16)	0.94761 (10)	0.39389 (8)	0.0166 (4)	
C58	0.89959 (16)	0.90683 (10)	0.45261 (7)	0.0152 (4)	
C59	0.78302 (16)	0.88017 (10)	0.46149 (7)	0.0157 (4)	
H59	0.7322	0.8861	0.4302	0.019*	
C60	0.74133 (16)	0.84458 (10)	0.51674 (7)	0.0166 (4)	
H60	0.6633	0.8268	0.5224	0.020*	
C61	0.81757 (16)	0.83602 (10)	0.56327 (7)	0.0172 (4)	
C62	0.93411 (16)	0.86335 (11)	0.55479 (8)	0.0195 (4)	
H62	0.9846	0.8580	0.5861	0.023*	
C63	0.97440 (16)	0.89826 (11)	0.49998 (8)	0.0178 (4)	
H63	1.0523	0.9163	0.4945	0.021*	
C64	0.67921 (18)	0.76180 (12)	0.62958 (8)	0.0271 (5)	
H64A	0.6727	0.7362	0.6698	0.041*	
H64B	0.6908	0.7193	0.6072	0.041*	
H64C	0.6024	0.8018	0.6186	0.041*	
C65	0.95912 (17)	1.18050 (11)	0.27986 (7)	0.0177 (4)	
C66	0.99348 (16)	1.26563 (11)	0.26387 (7)	0.0169 (4)	
C67	0.90216 (17)	1.33733 (11)	0.26628 (7)	0.0181 (4)	
H67	0.8189	1.3319	0.2785	0.022*	
C68	0.93296 (16)	1.41685 (11)	0.25075 (8)	0.0189 (4)	
H68	0.8709	1.4643	0.2523	0.023*	
C69	1.05760 (17)	1.42473 (11)	0.23278 (8)	0.0180 (4)	
C70	1.15106 (17)	1.35321 (11)	0.23154 (8)	0.0198 (4)	
H70	1.2349	1.3586	0.2206	0.024*	
C71	1.11896 (16)	1.27463 (11)	0.24661 (8)	0.0188 (4)	
H71	1.1813	1.2272	0.2453	0.023*	
C72	1.00517 (17)	1.57534 (11)	0.21247 (9)	0.0245 (4)	
H72A	1.0469	1.6229	0.2015	0.037*	
H72B	0.9628	1.5753	0.2495	0.037*	
H72C	0.9435	1.5786	0.1844	0.037*	
C73	0.87123 (16)	1.08805 (11)	0.17289 (7)	0.0169 (4)	
H73	0.9327	1.1208	0.1723	0.020*	
C74	0.79723 (16)	1.10346 (11)	0.12490 (8)	0.0172 (4)	
C75	0.70755 (17)	1.05289 (11)	0.12522 (8)	0.0205 (4)	
H75	0.6567	1.0610	0.0936	0.025*	
C76	0.69568 (17)	0.98979 (11)	0.17394 (8)	0.0219 (4)	
H76	0.6377	0.9545	0.1750	0.026*	
C77	0.77072 (17)	0.98031 (11)	0.22049 (8)	0.0198 (4)	
H77	0.7602	0.9393	0.2533	0.024*	
C78	0.82090 (17)	1.17471 (11)	0.07594 (8)	0.0193 (4)	
C79	1.13973 (16)	0.85400 (10)	0.27480 (7)	0.0156 (4)	
H79	1.0632	0.8348	0.2844	0.019*	
C80	1.24907 (16)	0.79606 (11)	0.26748 (7)	0.0156 (4)	
C81	1.36362 (16)	0.82537 (11)	0.25309 (7)	0.0173 (4)	
H81	1.4392	0.7884	0.2480	0.021*	
C82	1.36265 (17)	0.91065 (11)	0.24646 (8)	0.0193 (4)	
H82	1.4377	0.9316	0.2366	0.023*	
C83	1.24980 (16)	0.96386 (11)	0.25458 (7)	0.0177 (4)	
H83	1.2500	1.0209	0.2501	0.021*	
C84	1.23519 (17)	0.70559 (11)	0.27787 (7)	0.0172 (4)	

### Atomic displacement parameters (Å^2^)

**Table:**

	*U*^11^	*U*^22^	*U*^33^	*U*^12^	*U*^13^	*U*^23^
Zn1	0.01463 (11)	0.01483 (11)	0.01738 (11)	−0.00357 (8)	−0.00069 (8)	−0.00097 (8)
Zn2	0.01614 (11)	0.01688 (11)	0.01665 (11)	−0.00394 (8)	−0.00134 (8)	0.00155 (8)
Zn3	0.01497 (11)	0.01439 (11)	0.01705 (11)	−0.00305 (8)	−0.00064 (8)	−0.00048 (8)
O1	0.0182 (7)	0.0227 (7)	0.0227 (7)	−0.0074 (5)	0.0011 (5)	−0.0044 (5)
O2	0.0184 (7)	0.0254 (7)	0.0176 (7)	−0.0066 (5)	−0.0026 (5)	0.0002 (5)
O3	0.0288 (8)	0.0297 (8)	0.0163 (7)	−0.0102 (6)	−0.0039 (6)	0.0010 (6)
O4	0.0180 (7)	0.0222 (7)	0.0244 (7)	−0.0080 (5)	0.0000 (6)	−0.0009 (6)
O5	0.0181 (7)	0.0144 (6)	0.0288 (7)	−0.0035 (5)	−0.0015 (6)	−0.0014 (5)
O6	0.0178 (7)	0.0141 (6)	0.0287 (7)	−0.0043 (5)	−0.0002 (6)	−0.0025 (5)
O7	0.0256 (8)	0.0415 (9)	0.0287 (8)	−0.0206 (7)	−0.0077 (6)	0.0091 (7)
O8	0.0179 (7)	0.0176 (7)	0.0303 (8)	−0.0066 (5)	−0.0077 (6)	0.0027 (6)
O9	0.0195 (7)	0.0278 (7)	0.0166 (7)	−0.0064 (5)	−0.0022 (5)	0.0038 (5)
O10	0.0198 (7)	0.0244 (7)	0.0229 (7)	−0.0081 (6)	0.0013 (6)	−0.0032 (6)
O11	0.0238 (7)	0.0303 (7)	0.0142 (7)	−0.0113 (6)	−0.0039 (5)	0.0044 (6)
O12	0.0196 (7)	0.0251 (7)	0.0251 (7)	−0.0079 (6)	0.0011 (6)	−0.0010 (6)
O13	0.0209 (7)	0.0168 (7)	0.0261 (7)	−0.0048 (5)	−0.0014 (6)	−0.0023 (5)
O14	0.0211 (7)	0.0166 (7)	0.0301 (8)	−0.0047 (5)	−0.0024 (6)	−0.0028 (6)
O15	0.0203 (7)	0.0210 (7)	0.0440 (9)	−0.0067 (6)	−0.0133 (6)	0.0041 (6)
O16	0.0225 (7)	0.0347 (8)	0.0258 (8)	−0.0159 (6)	−0.0057 (6)	0.0079 (6)
O17	0.0176 (7)	0.0227 (7)	0.0233 (7)	−0.0071 (5)	0.0015 (5)	−0.0040 (6)
O18	0.0193 (7)	0.0253 (7)	0.0174 (7)	−0.0066 (5)	−0.0031 (5)	0.0019 (5)
O19	0.0248 (7)	0.0285 (7)	0.0143 (7)	−0.0104 (6)	−0.0018 (5)	−0.0005 (5)
O20	0.0196 (7)	0.0217 (7)	0.0240 (7)	−0.0092 (5)	0.0005 (6)	−0.0014 (6)
O21	0.0204 (7)	0.0145 (6)	0.0268 (7)	−0.0038 (5)	0.0002 (6)	−0.0029 (5)
O22	0.0191 (7)	0.0133 (6)	0.0380 (8)	−0.0040 (5)	−0.0010 (6)	−0.0013 (6)
O23	0.0192 (7)	0.0313 (8)	0.0250 (7)	−0.0100 (6)	−0.0036 (6)	0.0058 (6)
O24	0.0188 (7)	0.0172 (7)	0.0332 (8)	−0.0063 (5)	−0.0049 (6)	−0.0011 (6)
N1	0.0155 (8)	0.0151 (8)	0.0196 (8)	−0.0022 (6)	−0.0006 (6)	−0.0021 (6)
N2	0.0202 (9)	0.0230 (9)	0.0210 (9)	−0.0110 (7)	−0.0041 (7)	0.0057 (7)
N3	0.0163 (8)	0.0166 (8)	0.0143 (8)	−0.0036 (6)	−0.0013 (6)	−0.0019 (6)
N4	0.0187 (9)	0.0136 (8)	0.0280 (9)	−0.0049 (7)	−0.0031 (7)	−0.0025 (7)
N5	0.0172 (8)	0.0188 (8)	0.0145 (8)	−0.0049 (6)	−0.0014 (6)	−0.0010 (6)
N6	0.0223 (10)	0.0179 (9)	0.0324 (10)	−0.0046 (7)	−0.0048 (8)	−0.0018 (8)
N7	0.0171 (8)	0.0164 (8)	0.0181 (8)	−0.0033 (6)	−0.0003 (6)	−0.0010 (6)
N8	0.0182 (9)	0.0215 (9)	0.0206 (9)	−0.0075 (7)	−0.0040 (7)	0.0045 (7)
N9	0.0166 (8)	0.0155 (8)	0.0176 (8)	−0.0015 (6)	0.0009 (6)	−0.0039 (6)
N10	0.0194 (9)	0.0228 (9)	0.0218 (9)	−0.0052 (7)	−0.0026 (7)	0.0031 (7)
N11	0.0158 (8)	0.0158 (8)	0.0139 (7)	−0.0040 (6)	−0.0019 (6)	−0.0009 (6)
N12	0.0188 (9)	0.0146 (9)	0.0334 (10)	−0.0025 (7)	−0.0010 (7)	−0.0045 (7)
C1	0.0151 (9)	0.0138 (9)	0.0207 (10)	−0.0008 (7)	0.0016 (7)	−0.0036 (7)
C2	0.0161 (9)	0.0155 (9)	0.0192 (9)	−0.0017 (7)	0.0009 (7)	−0.0042 (7)
C3	0.0174 (9)	0.0147 (9)	0.0193 (10)	−0.0024 (7)	−0.0024 (7)	−0.0012 (7)
C4	0.0167 (9)	0.0183 (9)	0.0219 (10)	−0.0046 (7)	−0.0005 (8)	−0.0020 (8)
C5	0.0241 (10)	0.0155 (9)	0.0164 (9)	−0.0018 (7)	0.0004 (8)	−0.0020 (7)
C6	0.0205 (10)	0.0250 (10)	0.0199 (10)	−0.0017 (8)	−0.0058 (8)	−0.0061 (8)
C7	0.0148 (9)	0.0233 (10)	0.0216 (10)	−0.0034 (7)	−0.0013 (8)	−0.0047 (8)
C8	0.0406 (13)	0.0351 (12)	0.0193 (10)	−0.0185 (10)	−0.0015 (9)	0.0025 (9)
C9	0.0173 (10)	0.0179 (9)	0.0149 (9)	−0.0048 (7)	−0.0049 (7)	−0.0014 (7)
C10	0.0152 (9)	0.0160 (9)	0.0145 (9)	−0.0025 (7)	−0.0046 (7)	−0.0016 (7)
C11	0.0179 (9)	0.0157 (9)	0.0160 (9)	−0.0004 (7)	−0.0034 (7)	−0.0026 (7)
C12	0.0148 (9)	0.0207 (9)	0.0150 (9)	−0.0050 (7)	−0.0006 (7)	−0.0025 (7)
C13	0.0170 (9)	0.0161 (9)	0.0162 (9)	−0.0041 (7)	−0.0060 (7)	−0.0012 (7)
C14	0.0155 (9)	0.0159 (9)	0.0209 (10)	0.0006 (7)	−0.0028 (7)	−0.0046 (7)
C15	0.0128 (9)	0.0217 (10)	0.0199 (10)	−0.0036 (7)	−0.0012 (7)	−0.0023 (8)
C16	0.0241 (10)	0.0161 (9)	0.0276 (11)	−0.0014 (8)	−0.0042 (8)	−0.0037 (8)
C17	0.0145 (9)	0.0182 (9)	0.0207 (10)	−0.0042 (7)	0.0008 (7)	−0.0039 (7)
C18	0.0146 (9)	0.0182 (9)	0.0185 (9)	−0.0027 (7)	0.0014 (7)	−0.0029 (7)
C19	0.0184 (10)	0.0188 (9)	0.0203 (10)	−0.0037 (7)	−0.0022 (8)	−0.0036 (8)
C20	0.0193 (10)	0.0171 (9)	0.0249 (10)	−0.0075 (7)	−0.0016 (8)	−0.0023 (8)
C21	0.0204 (10)	0.0160 (9)	0.0182 (9)	−0.0022 (7)	−0.0008 (8)	−0.0004 (7)
C22	0.0217 (10)	0.0235 (10)	0.0164 (9)	−0.0072 (8)	−0.0006 (8)	−0.0014 (8)
C23	0.0165 (9)	0.0157 (9)	0.0150 (9)	−0.0055 (7)	−0.0032 (7)	0.0007 (7)
C24	0.0171 (9)	0.0186 (9)	0.0104 (8)	−0.0043 (7)	−0.0026 (7)	−0.0011 (7)
C25	0.0130 (9)	0.0204 (9)	0.0162 (9)	−0.0019 (7)	−0.0015 (7)	−0.0026 (7)
C26	0.0155 (9)	0.0212 (10)	0.0191 (10)	−0.0085 (7)	−0.0013 (7)	−0.0009 (7)
C27	0.0199 (10)	0.0141 (9)	0.0173 (9)	−0.0063 (7)	−0.0023 (7)	−0.0010 (7)
C28	0.0188 (10)	0.0183 (9)	0.0137 (9)	−0.0045 (7)	−0.0036 (7)	−0.0013 (7)
C29	0.0180 (10)	0.0141 (9)	0.0197 (10)	−0.0020 (7)	0.0003 (8)	−0.0021 (7)
C30	0.0170 (9)	0.0151 (9)	0.0161 (9)	−0.0032 (7)	−0.0002 (7)	−0.0034 (7)
C31	0.0154 (9)	0.0199 (9)	0.0210 (10)	−0.0046 (7)	−0.0031 (8)	−0.0021 (8)
C32	0.0164 (9)	0.0224 (10)	0.0172 (9)	−0.0024 (7)	−0.0054 (7)	−0.0030 (7)
C33	0.0205 (10)	0.0146 (9)	0.0129 (9)	−0.0035 (7)	−0.0007 (7)	−0.0008 (7)
C34	0.0130 (9)	0.0186 (9)	0.0213 (10)	−0.0054 (7)	−0.0027 (7)	−0.0023 (7)
C35	0.0188 (9)	0.0145 (9)	0.0157 (9)	−0.0018 (7)	−0.0064 (7)	−0.0015 (7)
C36	0.0297 (11)	0.0282 (11)	0.0153 (10)	−0.0064 (9)	−0.0056 (8)	0.0044 (8)
C37	0.0200 (10)	0.0216 (10)	0.0152 (9)	−0.0055 (8)	−0.0043 (8)	−0.0018 (7)
C38	0.0154 (9)	0.0197 (9)	0.0145 (9)	−0.0036 (7)	−0.0031 (7)	−0.0018 (7)
C39	0.0157 (9)	0.0242 (10)	0.0195 (10)	−0.0029 (8)	0.0006 (8)	−0.0026 (8)
C40	0.0186 (10)	0.0196 (10)	0.0233 (10)	0.0005 (8)	−0.0006 (8)	−0.0053 (8)
C41	0.0215 (10)	0.0174 (9)	0.0158 (9)	−0.0057 (7)	−0.0053 (8)	−0.0011 (7)
C42	0.0155 (9)	0.0232 (10)	0.0198 (10)	−0.0055 (7)	−0.0004 (7)	−0.0034 (8)
C43	0.0168 (9)	0.0197 (9)	0.0197 (10)	−0.0002 (7)	−0.0031 (7)	−0.0059 (8)
C44	0.0308 (11)	0.0176 (10)	0.0267 (11)	−0.0040 (8)	−0.0031 (9)	−0.0039 (8)
C45	0.0169 (9)	0.0201 (9)	0.0144 (9)	−0.0056 (7)	−0.0035 (7)	−0.0011 (7)
C46	0.0210 (10)	0.0206 (9)	0.0136 (9)	−0.0054 (8)	−0.0056 (7)	−0.0045 (7)
C47	0.0188 (10)	0.0254 (10)	0.0159 (9)	−0.0025 (8)	−0.0002 (8)	−0.0033 (8)
C48	0.0190 (10)	0.0264 (10)	0.0224 (10)	−0.0078 (8)	0.0041 (8)	0.0012 (8)
C49	0.0221 (10)	0.0199 (10)	0.0170 (9)	−0.0071 (8)	0.0001 (8)	0.0019 (7)
C50	0.0203 (10)	0.0205 (10)	0.0191 (10)	−0.0040 (8)	−0.0081 (8)	−0.0025 (8)
C51	0.0145 (9)	0.0175 (9)	0.0205 (10)	−0.0034 (7)	0.0000 (7)	−0.0029 (7)
C52	0.0161 (9)	0.0152 (9)	0.0175 (9)	−0.0015 (7)	0.0004 (7)	−0.0025 (7)
C53	0.0178 (10)	0.0194 (9)	0.0191 (10)	−0.0032 (7)	−0.0031 (8)	−0.0035 (7)
C54	0.0208 (10)	0.0183 (10)	0.0261 (11)	−0.0081 (8)	−0.0001 (8)	−0.0035 (8)
C55	0.0227 (10)	0.0126 (9)	0.0208 (10)	−0.0039 (7)	0.0008 (8)	−0.0010 (7)
C56	0.0171 (10)	0.0195 (9)	0.0164 (9)	−0.0043 (7)	0.0009 (7)	−0.0024 (7)
C57	0.0153 (9)	0.0129 (9)	0.0199 (10)	−0.0002 (7)	−0.0003 (7)	−0.0024 (7)
C58	0.0138 (9)	0.0130 (9)	0.0181 (9)	−0.0018 (7)	−0.0010 (7)	−0.0022 (7)
C59	0.0160 (9)	0.0138 (9)	0.0159 (9)	−0.0014 (7)	−0.0044 (7)	−0.0004 (7)
C60	0.0132 (9)	0.0164 (9)	0.0196 (10)	−0.0030 (7)	−0.0012 (7)	−0.0022 (7)
C61	0.0196 (10)	0.0151 (9)	0.0159 (9)	−0.0018 (7)	0.0009 (7)	−0.0028 (7)
C62	0.0164 (10)	0.0233 (10)	0.0183 (10)	−0.0020 (7)	−0.0048 (7)	−0.0032 (8)
C63	0.0130 (9)	0.0195 (9)	0.0216 (10)	−0.0041 (7)	−0.0009 (7)	−0.0047 (8)
C64	0.0304 (11)	0.0342 (12)	0.0176 (10)	−0.0156 (9)	0.0001 (8)	0.0007 (8)
C65	0.0185 (10)	0.0194 (9)	0.0150 (9)	−0.0042 (8)	−0.0048 (7)	−0.0011 (7)
C66	0.0175 (9)	0.0181 (9)	0.0155 (9)	−0.0037 (7)	−0.0035 (7)	−0.0030 (7)
C67	0.0140 (9)	0.0215 (10)	0.0189 (9)	−0.0027 (7)	−0.0002 (7)	−0.0053 (8)
C68	0.0158 (9)	0.0180 (9)	0.0221 (10)	−0.0004 (7)	−0.0018 (8)	−0.0047 (8)
C69	0.0187 (10)	0.0160 (9)	0.0198 (10)	−0.0043 (7)	−0.0033 (8)	−0.0027 (7)
C70	0.0131 (9)	0.0211 (10)	0.0245 (10)	−0.0043 (7)	−0.0003 (8)	−0.0028 (8)
C71	0.0167 (10)	0.0170 (9)	0.0216 (10)	−0.0001 (7)	−0.0036 (8)	−0.0037 (7)
C72	0.0236 (11)	0.0140 (9)	0.0347 (12)	−0.0013 (8)	−0.0061 (9)	−0.0032 (8)
C73	0.0136 (9)	0.0169 (9)	0.0189 (9)	−0.0026 (7)	0.0010 (7)	−0.0023 (7)
C74	0.0147 (9)	0.0181 (9)	0.0175 (9)	−0.0014 (7)	0.0010 (7)	−0.0031 (7)
C75	0.0185 (10)	0.0240 (10)	0.0183 (10)	−0.0021 (8)	−0.0008 (8)	−0.0049 (8)
C76	0.0210 (10)	0.0212 (10)	0.0254 (11)	−0.0085 (8)	0.0013 (8)	−0.0055 (8)
C77	0.0211 (10)	0.0168 (9)	0.0205 (10)	−0.0044 (7)	0.0009 (8)	−0.0018 (7)
C78	0.0179 (10)	0.0221 (10)	0.0160 (9)	−0.0016 (8)	0.0018 (7)	−0.0028 (7)
C79	0.0153 (9)	0.0169 (9)	0.0152 (9)	−0.0059 (7)	−0.0024 (7)	−0.0015 (7)
C80	0.0185 (9)	0.0171 (9)	0.0112 (9)	−0.0039 (7)	−0.0028 (7)	−0.0017 (7)
C81	0.0158 (9)	0.0188 (9)	0.0167 (9)	−0.0015 (7)	−0.0014 (7)	−0.0037 (7)
C82	0.0161 (9)	0.0227 (10)	0.0193 (10)	−0.0079 (7)	−0.0001 (7)	−0.0013 (8)
C83	0.0219 (10)	0.0148 (9)	0.0160 (9)	−0.0059 (7)	−0.0012 (7)	0.0000 (7)
C84	0.0188 (10)	0.0170 (9)	0.0153 (9)	−0.0028 (7)	−0.0042 (7)	−0.0018 (7)

### Geometric parameters (Å, °)

**Table:**

Zn1—O1	2.5181 (12)	C21—H21	0.9300
Zn1—O2	1.9631 (12)	C23—N3	1.339 (2)
Zn1—O5	1.9392 (12)	C23—C24	1.387 (2)
Zn1—N1	2.0793 (15)	C23—H23	0.9300
Zn1—N3	2.0561 (14)	C25—C24	1.393 (2)
Zn2—O9	1.9523 (12)	C25—C26	1.388 (2)
Zn2—O10	2.5931 (12)	C25—H25	0.9300
Zn2—O13	1.9317 (12)	C26—H26	0.9300
Zn2—N5	2.0536 (15)	C27—C26	1.378 (2)
Zn2—N7	2.0669 (14)	C27—H27	0.9300
Zn3—O17	2.4085 (12)	C28—C24	1.509 (2)
Zn3—O18	1.9987 (12)	C29—C30	1.491 (2)
Zn3—O21	1.9436 (12)	C30—C35	1.394 (2)
Zn3—N9	2.0840 (14)	C31—C30	1.391 (2)
Zn3—N11	2.0613 (14)	C31—C32	1.386 (2)
O2—C1	1.284 (2)	C31—H31	0.9300
O3—C5	1.364 (2)	C32—H32	0.9300
O3—C8	1.423 (2)	C33—C32	1.393 (2)
O4—C9	1.240 (2)	C33—C34	1.397 (2)
O5—C9	1.287 (2)	C34—H34	0.9300
O6—C13	1.363 (2)	C35—C34	1.384 (2)
O6—C16	1.432 (2)	C35—H35	0.9300
O7—C22	1.227 (2)	C36—O11	1.428 (2)
O8—C28	1.234 (2)	C36—H36A	0.9600
O9—C29	1.287 (2)	C36—H36B	0.9600
O10—C29	1.247 (2)	C36—H36C	0.9600
O11—C33	1.3653 (19)	C37—C38	1.497 (2)
O12—C37	1.245 (2)	C38—C39	1.387 (2)
O13—C37	1.289 (2)	C38—C43	1.399 (2)
O14—C41	1.367 (2)	C39—H39	0.9300
O14—C44	1.430 (2)	C40—C39	1.392 (2)
O15—C50	1.236 (2)	C40—C41	1.389 (2)
O16—C56	1.228 (2)	C40—H40	0.9300
O17—C57	1.251 (2)	C41—C42	1.396 (2)
O18—C57	1.279 (2)	C42—H42	0.9300
O19—C64	1.428 (2)	C43—C42	1.378 (2)
O20—C65	1.254 (2)	C43—H43	0.9300
O21—C65	1.282 (2)	C44—H44A	0.9600
O22—C69	1.366 (2)	C44—H44B	0.9600
O22—C72	1.431 (2)	C44—H44C	0.9600
O23—C78	1.234 (2)	C45—H45	0.9300
O24—C84	1.235 (2)	C46—C45	1.386 (2)
N1—C17	1.340 (2)	C46—C47	1.394 (2)
N1—C21	1.348 (2)	C46—C50	1.507 (2)
N2—C22	1.326 (2)	C47—H47	0.9300
N2—H2A	0.821 (19)	C48—C49	1.379 (2)
N2—H2B	0.836 (19)	C48—C47	1.388 (2)
N3—C27	1.352 (2)	C48—H48	0.9300
N4—C28	1.330 (2)	C49—H49	0.9300
N4—H4A	0.832 (18)	C51—C52	1.389 (2)
N4—H4B	0.85 (2)	C51—H51	0.9300
N5—C45	1.341 (2)	C52—C53	1.391 (2)
N5—C49	1.345 (2)	C53—H53	0.9300
N6—C50	1.333 (2)	C54—C53	1.392 (2)
N6—H6A	0.78 (2)	C54—C55	1.380 (2)
N6—H6B	0.86 (2)	C54—H54	0.9300
N7—C51	1.340 (2)	C55—H55	0.9300
N7—C55	1.347 (2)	C56—C52	1.511 (2)
N8—C56	1.333 (2)	C57—C58	1.490 (2)
N8—H8D	0.79 (2)	C59—C58	1.389 (2)
N8—H8E	0.87 (2)	C59—C60	1.395 (2)
N9—C77	1.350 (2)	C59—H59	0.9300
N10—C78	1.333 (2)	C60—H60	0.9300
N10—H10A	0.87 (2)	C61—O19	1.368 (2)
N10—H10B	0.84 (2)	C61—C60	1.389 (2)
N11—C79	1.338 (2)	C62—C61	1.394 (2)
N11—C83	1.353 (2)	C62—C63	1.378 (2)
N12—C84	1.332 (2)	C62—H62	0.9300
N12—H12A	0.83 (2)	C63—C58	1.396 (2)
N12—H12B	0.87 (2)	C63—H63	0.9300
C1—O1	1.254 (2)	C64—H64A	0.9600
C1—C2	1.489 (2)	C64—H64B	0.9600
C2—C3	1.391 (2)	C64—H64C	0.9600
C2—C7	1.399 (2)	C65—C66	1.492 (2)
C3—H3	0.9300	C66—C71	1.399 (2)
C4—C5	1.392 (2)	C67—C66	1.391 (2)
C4—C3	1.394 (2)	C67—H67	0.9300
C4—H4	0.9300	C68—C67	1.388 (2)
C6—C5	1.395 (2)	C68—C69	1.390 (2)
C6—C7	1.376 (2)	C68—H68	0.9300
C6—H6	0.9300	C70—C69	1.396 (2)
C7—H7	0.9300	C70—C71	1.379 (2)
C8—H8A	0.9600	C70—H70	0.9300
C8—H8B	0.9600	C71—H71	0.9300
C8—H8C	0.9600	C72—H72A	0.9600
C9—C10	1.496 (2)	C72—H72B	0.9600
C10—C11	1.397 (2)	C72—H72C	0.9600
C11—C12	1.378 (2)	C73—N9	1.339 (2)
C11—H11	0.9300	C73—C74	1.386 (2)
C12—H12	0.9300	C73—H73	0.9300
C13—C12	1.394 (2)	C74—C75	1.393 (2)
C13—C14	1.391 (2)	C74—C78	1.510 (2)
C14—C15	1.392 (2)	C75—H75	0.9300
C14—H14	0.9300	C76—C75	1.395 (2)
C15—C10	1.386 (2)	C76—C77	1.379 (2)
C15—H15	0.9300	C76—H76	0.9300
C16—H16A	0.9600	C77—H77	0.9300
C16—H16B	0.9600	C79—C80	1.385 (2)
C16—H16C	0.9600	C79—H79	0.9300
C17—H17	0.9300	C80—C81	1.397 (2)
C18—C17	1.387 (2)	C80—C84	1.504 (2)
C18—C19	1.390 (2)	C81—H81	0.9300
C18—C22	1.517 (2)	C82—C81	1.389 (2)
C19—C20	1.394 (2)	C82—C83	1.375 (2)
C19—H19	0.9300	C82—H82	0.9300
C20—C21	1.377 (2)	C83—H83	0.9300
C20—H20	0.9300		
			
O1—Zn1—O2	57.53 (5)	C31—C30—C35	118.79 (16)
O2—Zn1—N1	101.78 (5)	C35—C30—C29	120.46 (15)
O2—Zn1—N3	108.48 (5)	C30—C31—H31	119.4
O2—Zn1—C1	29.07 (5)	C32—C31—C30	121.29 (16)
O5—Zn1—O2	139.19 (5)	C32—C31—H31	119.4
O5—Zn1—N1	100.95 (5)	C31—C32—C33	119.24 (16)
O5—Zn1—N3	98.11 (5)	C31—C32—H32	120.4
O5—Zn1—C1	119.75 (5)	C33—C32—H32	120.4
N1—Zn1—C1	130.84 (6)	O11—C33—C32	124.34 (16)
N3—Zn1—N1	104.19 (6)	O11—C33—C34	115.38 (15)
N3—Zn1—C1	96.55 (5)	C32—C33—C34	120.24 (16)
O9—Zn2—O10	56.19 (5)	C35—C34—C33	119.60 (16)
O9—Zn2—N5	109.00 (5)	C35—C34—H34	120.2
O9—Zn2—N7	99.89 (5)	C33—C34—H34	120.2
O13—Zn2—O9	137.57 (6)	C30—C35—H35	119.6
O13—Zn2—N5	99.17 (6)	C34—C35—C30	120.83 (16)
O13—Zn2—N7	103.00 (5)	C34—C35—H35	119.6
N5—Zn2—N7	104.55 (6)	O11—C36—H36A	109.5
O17—Zn3—C57	29.18 (5)	O11—C36—H36B	109.5
O18—Zn3—O17	59.04 (4)	O11—C36—H36C	109.5
O18—Zn3—N9	98.24 (5)	H36A—C36—H36B	109.5
O18—Zn3—N11	109.87 (5)	H36A—C36—H36C	109.5
O18—Zn3—C57	29.86 (5)	H36B—C36—H36C	109.5
O21—Zn3—O17	96.15 (5)	O12—C37—O13	122.84 (17)
O21—Zn3—O18	140.66 (5)	O12—C37—C38	121.36 (16)
O21—Zn3—N9	102.61 (5)	O13—C37—C38	115.78 (16)
O21—Zn3—N11	97.49 (5)	C39—C38—C37	120.88 (16)
O21—Zn3—C57	119.88 (6)	C39—C38—C43	118.66 (16)
N9—Zn3—O17	157.26 (5)	C43—C38—C37	120.45 (16)
N9—Zn3—C57	128.09 (6)	C38—C39—C40	121.34 (17)
N11—Zn3—O17	86.98 (5)	C38—C39—H39	119.3
N11—Zn3—N9	102.99 (5)	C40—C39—H39	119.3
N11—Zn3—C57	99.72 (5)	C39—C40—H40	120.4
C1—O2—Zn1	102.95 (10)	C41—C40—C39	119.14 (17)
C5—O3—C8	117.49 (14)	C41—C40—H40	120.4
C9—O5—Zn1	111.31 (11)	O14—C41—C40	124.63 (16)
C13—O6—C16	118.41 (14)	O14—C41—C42	115.19 (16)
C29—O9—Zn2	105.34 (11)	C40—C41—C42	120.18 (17)
C33—O11—C36	117.70 (14)	C41—C42—H42	120.0
C37—O13—Zn2	111.31 (11)	C43—C42—C41	119.93 (17)
C41—O14—C44	118.14 (14)	C43—C42—H42	120.0
C57—O17—Zn3	81.06 (10)	C42—C43—C38	120.74 (17)
C57—O18—Zn3	99.02 (10)	C42—C43—H43	119.6
C61—O19—C64	117.23 (14)	C38—C43—H43	119.6
C65—O21—Zn3	109.51 (11)	O14—C44—H44A	109.5
C69—O22—C72	118.16 (14)	O14—C44—H44B	109.5
C17—N1—Zn1	118.23 (12)	O14—C44—H44C	109.5
C17—N1—C21	117.89 (15)	H44A—C44—H44B	109.5
C21—N1—Zn1	123.86 (12)	H44A—C44—H44C	109.5
C22—N2—H2B	124.0 (13)	H44B—C44—H44C	109.5
C22—N2—H2A	116.6 (13)	N5—C45—C46	123.50 (16)
H2B—N2—H2A	118.4 (18)	N5—C45—H45	118.3
C23—N3—Zn1	121.31 (12)	C46—C45—H45	118.3
C23—N3—C27	118.34 (15)	C45—C46—C47	117.95 (17)
C27—N3—Zn1	120.21 (11)	C45—C46—C50	116.88 (16)
C28—N4—H4A	122.3 (12)	C47—C46—C50	125.11 (16)
C28—N4—H4B	116.6 (13)	C46—C47—H47	120.7
H4A—N4—H4B	120.4 (18)	C48—C47—C46	118.69 (17)
C45—N5—Zn2	119.73 (12)	C48—C47—H47	120.7
C45—N5—C49	118.06 (15)	C47—C48—H48	120.2
C49—N5—Zn2	121.60 (12)	C49—C48—C47	119.64 (17)
C50—N6—H6A	115.9 (15)	C49—C48—H48	120.2
C50—N6—H6B	119.0 (14)	N5—C49—C48	122.15 (17)
H6B—N6—H6A	124 (2)	N5—C49—H49	118.9
C51—N7—Zn2	118.84 (12)	C48—C49—H49	118.9
C51—N7—C55	118.26 (15)	O15—C50—N6	123.07 (18)
C55—N7—Zn2	122.87 (12)	O15—C50—C46	118.91 (16)
C56—N8—H8E	123.6 (13)	N6—C50—C46	118.00 (17)
C56—N8—H8D	115.9 (15)	N7—C51—C52	123.23 (16)
H8E—N8—H8D	119.6 (19)	N7—C51—H51	118.4
C73—N9—Zn3	117.99 (12)	C52—C51—H51	118.4
C73—N9—C77	118.04 (15)	C51—C52—C53	118.03 (16)
C77—N9—Zn3	123.97 (12)	C51—C52—C56	116.96 (15)
C78—N10—H10A	122.7 (13)	C53—C52—C56	125.01 (16)
C78—N10—H10B	115.6 (14)	C52—C53—C54	119.05 (16)
H10A—N10—H10B	119.6 (19)	C52—C53—H53	120.5
C79—N11—C83	118.10 (15)	C54—C53—H53	120.5
C79—N11—Zn3	120.99 (11)	C53—C54—H54	120.5
C83—N11—Zn3	120.61 (11)	C55—C54—C53	119.09 (17)
C84—N12—H12B	123.2 (13)	C55—C54—H54	120.5
C84—N12—H12A	116.8 (15)	N7—C55—C54	122.33 (16)
H12B—N12—H12A	117.9 (19)	N7—C55—H55	118.8
O1—C1—Zn1	73.24 (10)	C54—C55—H55	118.8
O1—C1—O2	121.19 (16)	O16—C56—N8	123.52 (17)
O1—C1—C2	120.57 (16)	O16—C56—C52	119.29 (16)
O2—C1—Zn1	47.98 (8)	N8—C56—C52	117.19 (16)
O2—C1—C2	118.23 (15)	O17—C57—Zn3	69.76 (10)
C2—C1—Zn1	166.13 (13)	O17—C57—O18	120.86 (16)
C3—C2—C1	121.20 (16)	O17—C57—C58	120.06 (16)
C3—C2—C7	118.62 (16)	O18—C57—Zn3	51.12 (8)
C7—C2—C1	120.16 (16)	O18—C57—C58	119.07 (15)
C2—C3—C4	120.98 (16)	C58—C57—Zn3	170.01 (12)
C2—C3—H3	119.5	C59—C58—C57	120.79 (15)
C4—C3—H3	119.5	C59—C58—C63	119.04 (16)
C3—C4—H4	120.3	C63—C58—C57	120.11 (15)
C5—C4—C3	119.36 (16)	C58—C59—C60	120.75 (16)
C5—C4—H4	120.3	C58—C59—H59	119.6
O3—C5—C4	124.87 (16)	C60—C59—H59	119.6
O3—C5—C6	114.99 (16)	C59—C60—H60	120.3
C4—C5—C6	120.13 (17)	C61—C60—C59	119.34 (16)
C5—C6—H6	120.1	C61—C60—H60	120.3
C7—C6—C5	119.87 (17)	O19—C61—C60	124.66 (16)
C7—C6—H6	120.1	O19—C61—C62	115.07 (15)
C2—C7—H7	119.5	C60—C61—C62	120.25 (16)
C6—C7—C2	121.04 (17)	C61—C62—H62	120.1
C6—C7—H7	119.5	C63—C62—C61	119.86 (16)
O3—C8—H8A	109.5	C63—C62—H62	120.1
O3—C8—H8B	109.5	C58—C63—H63	119.6
O3—C8—H8C	109.5	C62—C63—C58	120.74 (16)
H8A—C8—H8B	109.5	C62—C63—H63	119.6
H8A—C8—H8C	109.5	O19—C64—H64A	109.5
H8B—C8—H8C	109.5	O19—C64—H64B	109.5
O4—C9—O5	123.06 (16)	H64A—C64—H64B	109.5
O4—C9—C10	121.34 (16)	O19—C64—H64C	109.5
O5—C9—C10	115.60 (15)	H64A—C64—H64C	109.5
C11—C10—C9	120.26 (15)	H64B—C64—H64C	109.5
C15—C10—C9	120.76 (16)	O20—C65—O21	122.42 (16)
C15—C10—C11	118.91 (16)	O20—C65—C66	121.08 (16)
C10—C11—H11	119.7	O21—C65—C66	116.49 (16)
C12—C11—C10	120.66 (16)	C67—C66—C71	118.74 (16)
C12—C11—H11	119.7	C67—C66—C65	120.88 (16)
C11—C12—C13	119.96 (17)	C71—C66—C65	120.38 (16)
C11—C12—H12	120.0	C66—C67—H67	119.4
C13—C12—H12	120.0	C68—C67—C66	121.24 (17)
O6—C13—C12	115.50 (15)	C68—C67—H67	119.4
O6—C13—C14	124.33 (16)	C67—C68—C69	119.25 (16)
C14—C13—C12	120.18 (16)	C67—C68—H68	120.4
C13—C14—C15	119.16 (16)	C69—C68—H68	120.4
C13—C14—H14	120.4	O22—C69—C68	124.29 (16)
C15—C14—H14	120.4	O22—C69—C70	115.54 (16)
C10—C15—C14	121.13 (17)	C68—C69—C70	120.16 (16)
C10—C15—H15	119.4	C69—C70—H70	120.0
C14—C15—H15	119.4	C71—C70—C69	119.99 (17)
O6—C16—H16A	109.5	C71—C70—H70	120.0
O6—C16—H16B	109.5	C66—C71—H71	119.7
O6—C16—H16C	109.5	C70—C71—C66	120.59 (16)
H16A—C16—H16B	109.5	C70—C71—H71	119.7
H16A—C16—H16C	109.5	O22—C72—H72A	109.5
H16B—C16—H16C	109.5	O22—C72—H72B	109.5
N1—C17—C18	123.41 (16)	O22—C72—H72C	109.5
N1—C17—H17	118.3	H72A—C72—H72B	109.5
C18—C17—H17	118.3	H72A—C72—H72C	109.5
C17—C18—C19	118.21 (16)	H72B—C72—H72C	109.5
C17—C18—C22	116.65 (15)	N9—C73—C74	123.42 (16)
C19—C18—C22	125.14 (16)	N9—C73—H73	118.3
C18—C19—C20	118.71 (17)	C74—C73—H73	118.3
C18—C19—H19	120.6	C73—C74—C75	118.29 (16)
C20—C19—H19	120.6	C73—C74—C78	116.66 (16)
C19—C20—H20	120.4	C75—C74—C78	125.05 (16)
C21—C20—C19	119.23 (17)	C74—C75—C76	118.54 (17)
C21—C20—H20	120.4	C74—C75—H75	120.7
N1—C21—C20	122.51 (16)	C76—C75—H75	120.7
N1—C21—H21	118.7	C75—C76—H76	120.3
C20—C21—H21	118.7	C77—C76—C75	119.37 (17)
O7—C22—N2	123.73 (17)	C77—C76—H76	120.3
O7—C22—C18	118.95 (16)	N9—C77—C76	122.30 (17)
N2—C22—C18	117.31 (16)	N9—C77—H77	118.8
N3—C23—C24	123.22 (16)	C76—C77—H77	118.8
N3—C23—H23	118.4	O23—C78—N10	123.34 (17)
C24—C23—H23	118.4	O23—C78—C74	119.05 (16)
C23—C24—C25	118.11 (16)	N10—C78—C74	117.61 (16)
C23—C24—C28	116.50 (15)	N11—C79—C80	123.44 (16)
C25—C24—C28	125.34 (16)	N11—C79—H79	118.3
C24—C25—H25	120.6	C80—C79—H79	118.3
C26—C25—C24	118.80 (16)	C79—C80—C81	117.96 (16)
C26—C25—H25	120.6	C79—C80—C84	116.83 (15)
C25—C26—H26	120.2	C81—C80—C84	125.17 (16)
C27—C26—C25	119.64 (16)	C80—C81—H81	120.6
C27—C26—H26	120.2	C82—C81—C80	118.84 (16)
N3—C27—C26	121.90 (16)	C82—C81—H81	120.6
N3—C27—H27	119.1	C81—C82—H82	120.3
C26—C27—H27	119.1	C83—C82—C81	119.44 (16)
O8—C28—N4	123.41 (17)	C83—C82—H82	120.3
O8—C28—C24	119.13 (15)	N11—C83—C82	122.22 (16)
N4—C28—C24	117.44 (16)	N11—C83—H83	118.9
O9—C29—C30	117.64 (15)	C82—C83—H83	118.9
O10—C29—O9	121.72 (16)	O24—C84—N12	123.03 (17)
O10—C29—C30	120.64 (16)	O24—C84—C80	119.32 (16)
C31—C30—C29	120.68 (16)	N12—C84—C80	117.64 (16)
			
O5—Zn1—O2—C1	−58.62 (13)	C1—C2—C7—C6	178.95 (16)
N1—Zn1—O2—C1	178.80 (10)	C3—C2—C7—C6	0.6 (3)
N3—Zn1—O2—C1	69.30 (11)	C5—C4—C3—C2	−0.4 (3)
O2—Zn1—O5—C9	−48.85 (14)	C3—C4—C5—O3	−179.89 (16)
N1—Zn1—O5—C9	73.99 (12)	C3—C4—C5—C6	0.9 (3)
N3—Zn1—O5—C9	−179.76 (11)	C7—C6—C5—O3	−179.91 (16)
C1—Zn1—O5—C9	−77.39 (12)	C7—C6—C5—C4	−0.6 (3)
O2—Zn1—N1—C17	160.84 (12)	C5—C6—C7—C2	−0.1 (3)
O2—Zn1—N1—C21	−17.59 (15)	O4—C9—C10—C11	−176.73 (16)
O5—Zn1—N1—C17	14.96 (13)	O4—C9—C10—C15	6.4 (3)
O5—Zn1—N1—C21	−163.47 (14)	O5—C9—C10—C11	3.4 (2)
N3—Zn1—N1—C17	−86.41 (13)	O5—C9—C10—C15	−173.43 (15)
N3—Zn1—N1—C21	95.16 (14)	C9—C10—C11—C12	−177.03 (15)
C1—Zn1—N1—C17	161.61 (11)	C15—C10—C11—C12	−0.1 (3)
C1—Zn1—N1—C21	−16.81 (17)	C10—C11—C12—C13	0.6 (3)
O2—Zn1—N3—C23	38.12 (14)	O6—C13—C12—C11	179.24 (15)
O2—Zn1—N3—C27	−137.51 (12)	C14—C13—C12—C11	−0.5 (3)
O5—Zn1—N3—C23	−173.27 (13)	O6—C13—C14—C15	−179.87 (16)
O5—Zn1—N3—C27	11.10 (13)	C12—C13—C14—C15	−0.2 (3)
N1—Zn1—N3—C23	−69.74 (13)	C13—C14—C15—C10	0.7 (3)
N1—Zn1—N3—C27	114.64 (13)	C14—C15—C10—C9	176.34 (16)
C1—Zn1—N3—C23	65.34 (13)	C14—C15—C10—C11	−0.6 (3)
C1—Zn1—N3—C27	−110.29 (13)	C19—C18—C17—N1	−1.4 (3)
O2—Zn1—C1—O1	−178.16 (17)	C22—C18—C17—N1	178.48 (16)
O2—Zn1—C1—C2	7.0 (5)	C17—C18—C19—C20	0.7 (3)
O5—Zn1—C1—O1	−38.16 (11)	C22—C18—C19—C20	−179.14 (17)
O5—Zn1—C1—O2	140.01 (10)	C17—C18—C22—O7	17.5 (3)
O5—Zn1—C1—C2	147.1 (5)	C17—C18—C22—N2	−161.67 (17)
N1—Zn1—C1—O1	−179.72 (9)	C19—C18—C22—O7	−162.65 (18)
N1—Zn1—C1—O2	−1.56 (13)	C19—C18—C22—N2	18.1 (3)
N1—Zn1—C1—C2	5.5 (5)	C18—C19—C20—C21	0.9 (3)
N3—Zn1—C1—O1	65.10 (10)	C19—C20—C21—N1	−2.0 (3)
N3—Zn1—C1—O2	−116.74 (11)	C24—C23—N3—C27	0.1 (2)
N3—Zn1—C1—C2	−109.7 (5)	C24—C23—N3—Zn1	−175.63 (12)
O13—Zn2—O9—C29	62.59 (13)	N3—C23—C24—C25	−0.4 (3)
N5—Zn2—O9—C29	−66.21 (12)	N3—C23—C24—C28	177.12 (15)
N7—Zn2—O9—C29	−175.47 (11)	C26—C25—C24—C23	0.2 (2)
O9—Zn2—O13—C37	47.40 (14)	C26—C25—C24—C28	−177.09 (16)
N5—Zn2—O13—C37	179.12 (12)	C24—C25—C26—C27	0.3 (3)
N7—Zn2—O13—C37	−73.52 (12)	N3—C27—C26—C25	−0.7 (3)
O9—Zn2—N5—C45	−19.92 (14)	O8—C28—C24—C23	10.9 (2)
O9—Zn2—N5—C49	151.00 (13)	O8—C28—C24—C25	−171.69 (17)
O13—Zn2—N5—C45	−167.74 (12)	N4—C28—C24—C23	−167.57 (16)
O13—Zn2—N5—C49	3.18 (14)	N4—C28—C24—C25	9.8 (3)
N7—Zn2—N5—C45	86.16 (13)	O9—C29—C30—C31	178.91 (16)
N7—Zn2—N5—C49	−102.92 (13)	O9—C29—C30—C35	2.1 (2)
O9—Zn2—N7—C51	−160.13 (13)	O10—C29—C30—C31	−1.3 (3)
O9—Zn2—N7—C55	17.74 (15)	O10—C29—C30—C35	−178.18 (16)
O13—Zn2—N7—C51	−16.11 (14)	C29—C30—C35—C34	177.52 (16)
O13—Zn2—N7—C55	161.75 (13)	C31—C30—C35—C34	0.6 (3)
N5—Zn2—N7—C51	87.12 (13)	C32—C31—C30—C29	−177.86 (16)
N5—Zn2—N7—C55	−95.01 (14)	C32—C31—C30—C35	−1.0 (3)
O18—Zn3—O17—C57	−0.80 (10)	C30—C31—C32—C33	0.5 (3)
O21—Zn3—O17—C57	146.61 (10)	O11—C33—C32—C31	178.25 (16)
N9—Zn3—O17—C57	0.99 (18)	C34—C33—C32—C31	0.3 (3)
N11—Zn3—O17—C57	−116.18 (10)	O11—C33—C34—C35	−178.77 (15)
O17—Zn3—O18—C57	0.78 (9)	C32—C33—C34—C35	−0.7 (3)
O21—Zn3—O18—C57	−56.88 (13)	C30—C35—C34—C33	0.2 (3)
N9—Zn3—O18—C57	−178.52 (10)	O12—C37—C38—C39	−2.3 (3)
N11—Zn3—O18—C57	74.39 (11)	O12—C37—C38—C43	178.60 (16)
O17—Zn3—O21—C65	−90.17 (11)	O13—C37—C38—C39	179.20 (16)
O18—Zn3—O21—C65	−43.40 (15)	O13—C37—C38—C43	0.1 (2)
N9—Zn3—O21—C65	76.90 (12)	C37—C38—C39—C40	−179.05 (16)
N11—Zn3—O21—C65	−177.93 (11)	C43—C38—C39—C40	0.1 (3)
C57—Zn3—O21—C65	−72.15 (12)	C37—C38—C43—C42	179.77 (16)
O17—Zn3—N9—C73	158.69 (12)	C39—C38—C43—C42	0.6 (3)
O17—Zn3—N9—C77	−20.9 (2)	C41—C40—C39—C38	−0.7 (3)
O18—Zn3—N9—C73	160.23 (12)	C39—C40—C41—O14	−179.09 (16)
O18—Zn3—N9—C77	−19.33 (14)	C39—C40—C41—C42	0.6 (3)
O21—Zn3—N9—C73	13.80 (13)	O14—C41—C42—C43	179.81 (15)
O21—Zn3—N9—C77	−165.76 (13)	C40—C41—C42—C43	0.1 (3)
N11—Zn3—N9—C73	−87.07 (13)	C38—C43—C42—C41	−0.7 (3)
N11—Zn3—N9—C77	93.37 (14)	C47—C46—C45—N5	0.2 (3)
C57—Zn3—N9—C73	159.30 (11)	C50—C46—C45—N5	−177.00 (15)
C57—Zn3—N9—C77	−20.27 (17)	C45—C46—C47—C48	0.3 (3)
O17—Zn3—N11—C79	84.93 (13)	C50—C46—C47—C48	177.26 (16)
O17—Zn3—N11—C83	−88.69 (13)	C45—C46—C50—O15	−10.4 (2)
O18—Zn3—N11—C79	29.46 (14)	C45—C46—C50—N6	168.43 (17)
O18—Zn3—N11—C83	−144.16 (12)	C47—C46—C50—O15	172.62 (17)
O21—Zn3—N11—C79	−179.26 (12)	C47—C46—C50—N6	−8.6 (3)
O21—Zn3—N11—C83	7.12 (13)	C49—C48—C47—C46	−0.8 (3)
N9—Zn3—N11—C79	−74.41 (13)	C47—C48—C49—N5	0.8 (3)
N9—Zn3—N11—C83	111.97 (13)	N7—C51—C52—C53	1.4 (3)
C57—Zn3—N11—C79	58.58 (13)	N7—C51—C52—C56	−178.01 (16)
C57—Zn3—N11—C83	−115.05 (13)	C51—C52—C53—C54	−0.5 (3)
O17—Zn3—C57—O18	−178.63 (17)	C56—C52—C53—C54	178.83 (16)
O18—Zn3—C57—O17	178.63 (17)	C55—C54—C53—C52	−0.9 (3)
O21—Zn3—C57—O17	−39.13 (11)	C53—C54—C55—N7	1.5 (3)
O21—Zn3—C57—O18	142.24 (10)	O16—C56—C52—C51	−14.7 (3)
N9—Zn3—C57—O17	−179.52 (9)	O16—C56—C52—C53	165.98 (18)
N9—Zn3—C57—O18	1.86 (13)	N8—C56—C52—C51	165.20 (17)
N11—Zn3—C57—O17	65.40 (10)	N8—C56—C52—C53	−14.1 (3)
N11—Zn3—C57—O18	−113.23 (10)	O17—C57—C58—C59	175.59 (16)
Zn1—O2—C1—O1	2.06 (19)	O17—C57—C58—C63	−1.6 (2)
Zn1—O2—C1—C2	−178.09 (12)	O18—C57—C58—C59	−3.6 (2)
C8—O3—C5—C4	7.7 (3)	O18—C57—C58—C63	179.17 (15)
C8—O3—C5—C6	−173.03 (16)	C60—C59—C58—C57	−177.83 (15)
Zn1—O5—C9—O4	3.7 (2)	C60—C59—C58—C63	−0.6 (2)
Zn1—O5—C9—C10	−176.46 (11)	C58—C59—C60—C61	0.1 (3)
C16—O6—C13—C12	178.37 (15)	C60—C61—O19—C64	12.2 (2)
C16—O6—C13—C14	−2.0 (2)	C62—C61—O19—C64	−169.53 (16)
Zn2—O9—C29—O10	−0.3 (2)	O19—C61—C60—C59	178.68 (16)
Zn2—O9—C29—C30	179.44 (12)	C62—C61—C60—C59	0.5 (3)
C36—O11—C33—C32	4.8 (2)	C63—C62—C61—O19	−178.98 (15)
C36—O11—C33—C34	−177.21 (15)	C63—C62—C61—C60	−0.6 (3)
Zn2—O13—C37—O12	−4.3 (2)	C61—C62—C63—C58	0.1 (3)
Zn2—O13—C37—C38	174.17 (11)	C62—C63—C58—C57	177.72 (16)
C44—O14—C41—C40	2.5 (3)	C62—C63—C58—C59	0.5 (3)
C44—O14—C41—C42	−177.25 (15)	O21—C65—C66—C67	170.90 (16)
Zn3—O17—C57—O18	1.24 (15)	O21—C65—C66—C71	−9.7 (2)
Zn3—O17—C57—C58	−177.97 (15)	O20—C65—C66—C67	−8.0 (3)
Zn3—O18—C57—O17	−1.50 (18)	O20—C65—C66—C71	171.43 (16)
Zn3—O18—C57—C58	177.72 (13)	C65—C66—C71—C70	179.70 (16)
Zn3—O21—C65—O20	2.3 (2)	C67—C66—C71—C70	−0.9 (3)
Zn3—O21—C65—C66	−176.59 (11)	C68—C67—C66—C65	−179.13 (16)
C72—O22—C69—C68	−3.6 (3)	C68—C67—C66—C71	1.4 (3)
C72—O22—C69—C70	176.33 (16)	C69—C68—C67—C66	−0.4 (3)
C21—N1—C17—C18	0.4 (3)	C67—C68—C69—O22	178.65 (16)
Zn1—N1—C17—C18	−178.16 (13)	C67—C68—C69—C70	−1.3 (3)
Zn1—N1—C21—C20	179.77 (13)	C71—C70—C69—O22	−178.09 (16)
C17—N1—C21—C20	1.3 (3)	C71—C70—C69—C68	1.8 (3)
Zn1—N3—C27—C26	176.25 (13)	C69—C70—C71—C66	−0.8 (3)
C23—N3—C27—C26	0.5 (2)	C74—C73—N9—C77	0.9 (3)
Zn2—N5—C45—C46	171.01 (13)	C74—C73—N9—Zn3	−178.69 (13)
C49—N5—C45—C46	−0.2 (3)	N9—C73—C74—C75	−1.7 (3)
Zn2—N5—C49—C48	−171.37 (14)	N9—C73—C74—C78	178.28 (16)
C45—N5—C49—C48	−0.3 (3)	C73—C74—C75—C76	0.6 (3)
Zn2—N7—C51—C52	177.20 (13)	C78—C74—C75—C76	−179.40 (16)
C55—N7—C51—C52	−0.8 (3)	C73—C74—C78—O23	18.3 (3)
Zn2—N7—C55—C54	−178.59 (13)	C73—C74—C78—N10	−161.14 (17)
C51—N7—C55—C54	−0.7 (3)	C75—C74—C78—O23	−161.71 (18)
Zn3—N9—C77—C76	−179.39 (13)	C75—C74—C78—N10	18.8 (3)
C73—N9—C77—C76	1.0 (3)	C77—C76—C75—C74	1.2 (3)
Zn3—N11—C79—C80	−173.43 (13)	C75—C76—C77—N9	−2.1 (3)
C83—N11—C79—C80	0.4 (2)	N11—C79—C80—C81	0.0 (3)
Zn3—N11—C83—C82	173.53 (13)	N11—C79—C80—C84	177.85 (15)
C79—N11—C83—C82	−0.3 (2)	C79—C80—C81—C82	−0.4 (2)
Zn1—C1—C2—C3	−11.2 (6)	C84—C80—C81—C82	−178.06 (16)
Zn1—C1—C2—C7	170.4 (4)	C79—C80—C84—O24	7.0 (2)
O1—C1—C2—C3	174.56 (16)	C79—C80—C84—N12	−173.62 (16)
O1—C1—C2—C7	−3.8 (3)	C81—C80—C84—O24	−175.31 (17)
O2—C1—C2—C3	−5.3 (2)	C81—C80—C84—N12	4.1 (3)
O2—C1—C2—C7	176.36 (15)	C83—C82—C81—C80	0.5 (3)
C1—C2—C3—C4	−178.65 (16)	C81—C82—C83—N11	−0.1 (3)
C7—C2—C3—C4	−0.3 (3)		

### Hydrogen-bond geometry (Å, °)

**Table:**

*D*—H···*A*	*D*—H	H···*A*	*D*···*A*	*D*—H···*A*
N2—H2A···O24^i^	0.82 (2)	2.16 (2)	2.966 (2)	168.8 (2)
N2—H2B···O4^ii^	0.84 (2)	2.16 (2)	2.991 (2)	176.6 (2)
N4—H4A···O17^iii^	0.83 (2)	2.08 (2)	2.903 (2)	169.4 (2)
N4—H4B···O16^iv^	0.85 (2)	2.59 (2)	3.152 (2)	125.0 (2)
N4—H4B···O19^v^	0.85 (2)	2.52 (2)	3.225 (2)	141.3 (2)
N6—H6A···O23^vi^	0.78 (2)	2.40 (2)	3.073 (2)	146 (2)
N6—H6B···O10^vii^	0.86 (2)	2.11 (2)	2.959 (2)	170.3 (2)
N8—H8D···O8^iv^	0.79 (2)	2.13 (2)	2.909 (2)	166 (2)
N8—H8E···O20^viii^	0.87 (2)	2.19 (2)	3.058 (2)	174.8 (2)
N10—H10A···O12^ix^	0.87 (2)	2.21 (2)	3.077 (2)	172 (2)
N10—H10B···O15^vi^	0.84 (2)	2.10 (2)	2.917 (2)	162.8 (2)
N12—H12A···O11^iii^	0.83 (2)	2.55 (2)	3.268 (2)	145 (2)
N12—H12B···O1^iii^	0.87 (2)	2.07 (2)	2.940 (2)	171.9 (2)
C4—H4···O14^x^	0.93	2.45	3.331 (2)	158
C19—H19···O4^ii^	0.93	2.36	3.175 (2)	146
C25—H25···O17^iii^	0.93	2.41	3.284 (2)	157
C27—H27···O7^xi^	0.93	2.47	3.214 (2)	138
C34—H34···O22^xii^	0.93	2.51	3.329 (2)	148
C35—H35···O15^xiii^	0.93	2.45	3.304 (2)	152
C47—H47···O10^vii^	0.93	2.27	3.146 (2)	156
C53—H53···O20^viii^	0.93	2.31	3.172 (2)	154
C59—H59···O8^xi^	0.93	2.58	3.390 (2)	146
C60—H60···O6	0.93	2.42	3.282 (2)	153
C75—H75···O12^ix^	0.93	2.39	3.212 (2)	147
C81—H81···O1^iii^	0.93	2.44	3.306 (2)	156

Symmetry codes: (i) *x*−1, *y*, *z*; (ii) −*x*, −*y*+1, −*z*+1; (iii) −*x*+2, −*y*+1, −*z*+1; (iv) −*x*+1, −*y*, −*z*+2; (v) *x*, *y*−1, *z*; (vi) −*x*+1, −*y*+2, −*z*+1; (vii) −*x*, −*y*+1, −*z*+2; (viii) *x*, *y*−1, *z*+1; (ix) *x*, *y*+1, *z*−1; (x) −*x*, −*y*, −*z*+2; (xi) −*x*+1, −*y*+1, −*z*+1; (xii) −*x*+2, −*y*+2, −*z*+1; (xiii) −*x*+1, −*y*+1, −*z*+2.
